# Artificial intelligence-assisted diagnosis of early gastric cancer: present practice and future prospects

**DOI:** 10.1080/07853890.2025.2461679

**Published:** 2025-02-10

**Authors:** Changda Lei, Wenqiang Sun, Kun Wang, Ruixia Weng, Xiuji Kan, Rui Li

**Affiliations:** a Department of Gastroenterology, The First Affiliated Hospital of Soochow University, Suzhou, China; b Suzhou Medical College, Soochow University, Suzhou, China; c Department of Neonatology, Children’s Hospital of Soochow University, Suzhou, China

**Keywords:** Early gastric cancer, artificial intelligence, deep learning, convolutional neural networks

## Abstract

Gastric cancer (GC) occupies the first few places in the world among tumors in terms of incidence and mortality, causing serious harm to human health, and at the same time, its treatment greatly consumes the health care resources of all countries in the world. The diagnosis of GC is usually based on histopathologic examination, and it is very important to be able to detect and identify cancerous lesions at an early stage, but some endoscopists’ lack of diagnostic experience and fatigue at work lead to a certain rate of under diagnosis. The rapid and striking development of Artificial intelligence (AI) has helped to enhance the ability to extract abnormal information from endoscopic images to some extent, and more and more researchers are applying AI technology to the diagnosis of GC. This initiative has not only improved the detection rate of early gastric cancer (EGC), but also significantly improved the survival rate of patients after treatment. This article reviews the results of various AI-assisted diagnoses of EGC in recent years, including the identification of EGC, the determination of differentiation type and invasion depth, and the identification of borders. Although AI has a better application prospect in the early diagnosis of ECG, there are still major challenges, and the prospects and limitations of AI application need to be further discussed.

## 1. Introduction

Gastric cancer (GC) is a multifactorial malignant tumor associated with diet, environment, and heredity, with highly aggressive and heterogeneous characteristics [1,2]. Its incidence is the fourth most common cancer in the world [3]. The median survival of patients with advanced GC is less than 1 year, and some studies have shown that patients with progressive GC have a survival rate of less than 20% at 5 years after surgery [4,5]. Early gastric cancer (EGC) means that the tumor tissue is confined to the mucosa and submucosa, regardless of whether there are metastases in lymph nodes or not, and its 5-year survival rate can be as high as 90% or more after active treatment. Thus, early diagnosis and appropriate treatment are the key measures to reduce the mortality rate of GC [6,7].

Gastroscopy is still the most common and efficient test for the diagnosis of GC and EGC [8]. A Meta-analysis that included studies from 22 countries showed that 622 out of 6961 patients with GC were underdiagnosed, a rate of 8.94%[9]. There are numerous reasons for missed diagnosis of GC and EGC, including endoscopists’ inadequate knowledge of the lesion, incomplete understanding of gastric anatomy, and biopsy errors [10–12]. In response to the human subjective factors that lead to the missed diagnosis of GC, Japanese experts have recommended the implementation of precision gastroscopy to reduce the rate of missed diagnosis [13]. Studies have shown that image enhancement techniques such as Narrow Band Imaging (NBI), Magnifying endoscopy (ME), Blue laser imaging (BLI) and Linked color imaging (LCI) are more beneficial to the early detection of GC than conventional white light capabilities [14]. However, advanced image enhancement technologies cannot be deployed in most underdeveloped regions, and the long time required for endoscopists to learn such techniques and the differences in learning ability between different endoscopists lead to differences in final diagnostic results [15]. Therefore, in addition to the assistance of such image enhancement techniques, it is particularly important to seek new, simple, fast, and accurate aids to help endoscopists identify lesions.

**Figure 1.**
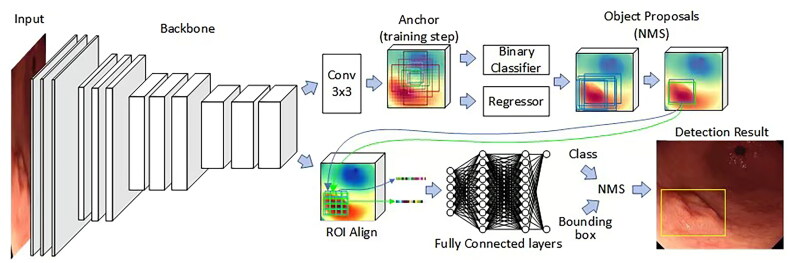
Structure of the classical detection model fast-cnns. Convolutional layers are used to computationally obtain different feature maps, the convolutional layers located at the beginning of the network architecture are used to detect low-level semantic features such as edges and curves, while the convolutional layers located deeper into the model architecture are used to learn more abstract semantic features. An activation function is applied to the convolution results to obtain a feature map. The pooling layer serves to reduce the computational burden on the network by decreasing the spatial dimensions of the feature map and the number of parameters in the network without losing information. By stacking several convolutional and pooling layers, feature maps with high-level semantic information can be obtained gradually. One or more fully connected layers follow the convolutional and pooling layers for integrated extraction of features to enhance the feature representation of the model. In a convolutional neural network, each neuron of the feature map is connected to the region of a neighboring neuron in the previous layer, such a neighborhood is called the neuron’s receptive field. When generating a feature map at a certain level, the convolution kernel of that level is shared by all spatial locations of the input, and this weight sharing can effectively reduce the parameters of the network and reduce the risk of overfitting of the network. Complete feature information is obtained by setting different convolution kernels at different levels. The last layer of the convolutional neural network is the output layer, which can usually be used to obtain the best parameters for a particular task and output predictions by minimizing the loss function defined on that task. (Endoscopic gastric mucosal morphology was obtained from the First Affiliated Hospital of Soochow University, with written informed consent from the patient and a statement of consent for publication.) (Figure drawing by Visio 2024).

Artificial intelligence (AI) is a technology that simulates human intelligence through computer programs. Machine learning, as an important part of AI, is able to construct constantly iterative models based on data to improve the ability to solve specific problems [16–18]. Deep learning is a branch of machine learning that develops algorithms based on artificial neural networks that mimic the architecture of human neurons to effectively extract high-level semantic information as well as richly detailed information [19,20]. Deep learning structures include deep neural networks, deep confidence networks, and convolutional neural networks (CNN). Among them, CNN performs better in image recognition and is therefore widely used in computer vision [21,22] (Figure 1). Currently, AI has made a series of advances in the field of medical image recognition. for example, in diabetic retinopathy, skin cancer, intracranial aneurysm, and breast disease, all of which have shown efficient diagnostic ability [23–27]. Similarly, AI also plays a huge role in diagnosing digestive disorders, such as advanced tumors of the colon, squamous cell carcinoma of the esophagus, and focal liver lesions [28–30]. Although some studies on AI for detecting gastrointestinal tumors have already appeared, the existing research directions are further enriched with the development of science and technology.

Based on the current situation, we searched the database for a large number of journals closely related to the research direction of this paper, from which we prioritized high-quality articles with reliable research design and high citation counts. In summary, this paper not only enumerates in detail the role of AI in EGC detection, but also its application in the nature judgment and boundary recognition of EGC, as well as the shortcomings of AI and the future development prospects [18,22,31].

## 2. AI in early gastric cancer detection

As mentioned earlier, the prognosis of EGC is significantly better than that of progressive GC, so timely detection and accurate diagnosis of EGC has become a key strategy to prolong the survival and improve the quality of life of patients. Currently, AI technology only provides endoscopists with important references and hints when diagnosing diseases, rather than completely replacing experts in diagnosis, let alone shaking the status of pathological biopsy as the “gold standard” [32]. If reliable clinical data and high-quality endoscopic image data can be provided, the introduction of AI technology built in such a way to the daily diagnosis and treatment work can greatly save physicians’ working time.

### 2.1. Based on white light endoscopy

General gastroscopy, also known as white light endoscopy (WLE), is still the most widely used test to screen for GC, which is mediated by a miniature image sensor that transmits an image to a television monitor, which visualizes the surface of the gastric mucosa with a white light source [8]. Several studies have reported the role of AI in detecting EGC in WLE (Table 1).

**Table 1.** AI in white light endoscopy for early gastric cancer detection.

Authors	Year	Country	Performance
Yuan [33]	2022	China	Accuracy:93.5%,Sensitivity:59.2%, Specificity:99.3%
Wu [34]	2019	China	Accuracy:92.5%,Sensitivity:94.0%, Specificity:91.0%, Positive predictive value:91.3%, Negative predictive value:93.8%
Feng [35]	2022	China	Internal test: Accuracy:88.60%, Sensitivity:88.08%, Specificity:89.05% External text: Accuracy:92.07%, Sensitivity:92.08%, Specificity;92.05%
Zhang [36]	2021	China	Accuracy:78.7%, Sensitivity:36.8%, Specificity:91.2%
Kim [37]	2024	South Korea	Accuracy:60.83%, Sensitivity:67.20%, Specificity:71.22%
Nam JY [38]	2022	South Korea	Internal test: Accuracy:94%, Sensitivity:90%, Specificity:93% External text: Accuracy:89%, Sensitivity:77%, Specificity:79%
Hirasawa [39]	2018	Japan	Sensitivity:92.6%
Yao [40]	2022	China	External text (1): Accuracy:85.15%, Sensitivity:85.36%, Specificity:84.41%; External text (2): Accuracy:86.02%, Sensitivity:83.02%, Specificity:92.21%
Oura [41]	2022	Japan	Accuracy:93.2%
Wu [42]	2021	China	Accuracy:84.7%, Specificity:100%, Sensitivity:84.3%
Luo [43]	2019	China	Accuracy:92.7%, Specificity:94.6%, Sensitivity:91.3%
Tang [44]	2020	China	Accuracy:87.5%, Sensitivity:95.58%, Specificity:81.7%
Dong [45]	2023	China	Internal Video Test: Accuracy:81.10% External Video Test: Accuracy:88.24%

AI: artificial intelligence.

A large-scale study of WLE-based diagnosis of gastric lesions included 29,809 images of 8,947 patients containing EGCs, advanced GCs, submucosal tumors, polyps, peptic ulcers, erosions, and lesion-free gastric mucosa. The overall accuracy of the system for EGC diagnosis was 93.5%, which was comparable to the diagnostic ability of senior endoscopists. With the assistance of the system, the accuracy of senior and junior endoscopists in the diagnosis of EGC was increased to 94.9% and 94.1%, respectively, which was a significant diagnostic aid [33]. Wu et al. developed an EGC detection system based on deep convolutional neural networks (DCNN). Diagnostic performance was significantly better than all levels of endoscopists, achieving 92.5% accuracy, 94.0% sensitivity, 91.0% specificity, 91.3% positive predictive value, and 93.8% negative predictive value, and the system has an automated function for monitoring blind spots during gastroscopy, which suggests anatomical areas that are not reached by the field of view when the endoscopist is examining them [34]. The DCNN-based system developed by Feng et al. collected 3400 EGC images and 8600 benign images to train the DCNN to detect EGCs, and the results showed that in the internal test set, the diagnostic sensitivity, specificity, and accuracy of the DCNN were 88.08%, 89.05%, and 88.60%, respectively, with a significantly higher sensitivity and accuracy than that of all endoscopists. In the external test set, the diagnostic sensitivity, specificity, and accuracy were 92.08%, 92.05%, and 92.07%, respectively, which were significantly higher than that of all endoscopists in terms of sensitivity and specificity, and significantly higher than that of endoscopists of lower seniority in terms of accuracy. In addition, the study analyzed the reasons for false-positive and false-negative DCNN diagnoses, such as mucus, bleeding, vascularity, and H. pylori infection [35]. Zhang et al. developed and trained a DCNN diagnostic system based on ResNet34 residual network structure and DeepLabv3 structure, which incorporated a total of 21217 gastroscopy images of peptic ulcer, EGC and high-grade intraepithelial neoplasia, advanced gastric carcinoma, gastric submucosal tumors, and normal gastric mucosa without lesions, constructed and trained a DCNN diagnostic system based on ResNet34 residual network architecture and DeepLabv3 architecture for CNN diagnosis system. The trained CNN was evaluated using a test dataset of 1091 images and achieved accuracy, sensitivity, specificity, positive predictive value, and negative predictive value of 78.7%, 36.8%, 91.2%, 55.4%, and 82.9%, respectively, with specificity and positive predictive value significantly higher than that of the endoscopist of 86.7%, 41.7% [36] Kim et al. built a new CNN to detect and classify benign gastric lesions, precancerous lesions, and malignant tumors using 10,181 white light endoscopic images from 2606 patients. The system achieved 100% EGC detection rate with negative predictive value, positive predictive value, specificity, sensitivity, and accuracy of 85.88%, 45.42%, 71.22%, 67.20%, and 60.83%, respectively [37]. A study from South Korea included 1,366 patients with gastric mucosal lesions from 2 referral centers and used a representative white light endoscopic image of each patient to compare the diagnostic ability of the AI-DDX system with that of physicians of different years of experience. The AI-DDx model demonstrated good diagnostic performance with an AUC of 0.92 in the internal test set for early gastric cancer, significantly higher than all endoscopists, and an AUC of 0.86 in the external test set, significantly higher than both low and middle-aged practitioners [38]. T. Hirasawa et al. constructed a CNN-based diagnostic system based on a single multibox detector and trained it using 13,584 endoscopic images of gastric cancer. An independent test set of 2296 stomach images collected from 69 patients with gastric cancer lesions was applied to the constructed CNN, and the model achieved an overall sensitivity of 92.2% [39]. Yao et al. utilized more than 40,000 gastroscopy images of 1653 patients as the training set for the AI system “EGC-YOLO”, and endoscopy images from two other hospitals as the external validation test set, and the results of the study showed that the accuracy, sensitivity, specificity, and positive predictive value of the external test set 1 were 85.15%, 86.02%, 85.36%, 83.02%, and 84.41%, 92.02% in the external test set 2, and 83.02% in the external test set 2. The results showed that the accuracy, sensitivity, specificity and positive predictive value in the external test set 1 were 85.15%, 86.02%, 85.36%, 83.02%, and in the external test set 2, the accuracy, sensitivity, specificity and positive predictive value were 84.41%, 92.21%, 95.22%, 95.65%, respectively, which demonstrated the ability of the system to efficiently, accurately and rapidly detect early gastric cancer [40]. Oura et al. developed and validated a lesion detection system from 12,977 static gastroscopy images from 855 cancer patients, which had a 93.2% accuracy in diagnosing early gastric cancer [41]. Wu et al. developed an artificial intelligence system, ENDOANGEL, using DCNN combined with deep reinforcement learning, and verified in a randomized controlled study based on patients who underwent gastroscopy at five hospitals that ENDOANGEL was able to reliably diagnose early gastric cancer with an accuracy, sensitivity, and specificity of 84.7%, 100%, and 84.3%, respectively [42]. The system provides effective training for inexperienced endoscopists to improve their observation skills during examinations.

Luo et al. developed and validated a gastrointestinal artificial intelligence diagnostic system for diagnosing upper gastrointestinal cancers. In this multicenter study, the authors developed the system using Deep Lab’s V3+ concept. The results showed that the model diagnosed EGC with an accuracy, sensitivity, and specificity of 92.7%, 94.6%, and 91.3%, respectively [43]. This is a large-scale study of AI for diagnosing upper gastrointestinal cancers. The real-time DCNN system developed by Tang et al. utilized 35,823 images from 1085 patients to construct the training model and evaluated the diagnostic capability of the system in an internal validation dataset (9,417 images from 279 patients) and an external validation set (a total of 1,514 images from three other hospitals), which showed that the system’s accuracy in the different validation sets was 85.1%- 91.2%, sensitivity of 85.9%-95.5%, specificity of 81.7%-90.3%, and AUC of 0.887-0.940 [44]. AI systems have previously lacked interpretability, affecting their trust and acceptance in the clinical domain. Dong et al. developed ENDOANGEL-ED, an interpretable AI, which was trained and tested using images of 3,279 patients and 296 videos of focal lesions from eight hospitals. ENDOANGEL-ED performed well in the image and video tests, and its accuracy in the human-computer comparison was significantly higher than endoscopists. The AI not only improves endoscopists’ diagnostic ability, but also increases their trust and acceptance. In summary, ENDOANGEL-ED is a high-performance, real-time interpretable AI system with high clinical credibility and acceptance [45].

Although WLE-based AI systems have been frequently developed, there are some shortcomings in WLE itself, such as the lack of observation of subtle local changes in the mucosa, and in some cases it is difficult to show subtle changes in the color and morphology of the diseased mucosa, which is often difficult to differentiate from benign lesions. For inexperienced endoscopists, it is easy to miss the diagnosis of poorly characterized and small EGCs under WLE [46,47]. In recent years, with the continuous advancement of optical imaging technology, new endoscopic modalities have been gradually developed.

### 2.2. Based on image-enhanced endoscopy (IEE)

Among the new image-enhanced endoscopes presented in recent years, Narrow Band Imaging (NBI) and Blue Light Imaging (BLI) have received a lot of attention due to their unique imaging characteristics. NBI improves the detection of EGCs by filtering out some wavelengths of the visible spectrum and retaining only narrow-band light in specific wavelength bands, which makes submucosal blood vessels and surface structures clearer in the image, while BLI improves the contrast of the mucosal surface by increasing the proportion of blue light, which makes the lesion area more conspicuous in the image and improves the diagnostic accuracy of EGCs [48,49]. Magnifying endoscopy (ME) has been widely used to improve the diagnostic efficacy of EGC, as it is able to clearly visualize the microstructure and microvasculature of the gastric mucosal layer [50]. Magnified endoscopy combined with narrow-band imaging (ME-NBI), as an optical technique of great significance in endoscopic diagnosis and treatment, has been shown to be of high value in the diagnosis of EGC. Providing ME-NBI image data to CNNs for diagnosing diseases is a promising endeavor [51,52]. Several studies have reported the role of AI in detecting EGC in ME-NBI (Table 2).

**Table 2.** Application of AI in early gastric cancer detection under IEE.

Authors	Year	Country	Endoscopic model	Performance
Tang [53]	2022	China	ME-NBI	Accuracy:93.5%, Sensitivity:59.2%, Specificity:99.3%
Li [54]	2020	China	ME-NBI	Accuracy:90.91%, Sensitivity:91.18%, Specificity:90.64%
Horiuchi [55]	2020	Japan	ME-NBI	Accuracy:85.1%, Sensitivity:87.4%, Specificity:82.8%
He [56]	2022	China	ME-NBI	Internal images test:Accuracy:88.44% External images test:Accuracy:90.49% Internal videos test:Accuracy:90.32%
Ueyama [57]	2021	Japan	ME-NBI	Accuracy:98.7%, Sensitivity:98%, Specificity:100%
Kanesaka [58]	2017	Japan	ME-NBI	Accuracy:96.3%, Sensitivity:96.7%, Specificity:95%
Hu [59]	2021	China	ME-NBI	Internal test:AUC:0.808, External test:AUC:0.813
Liu [60]	2022	China	ME-NBI	Accuracy:90.8%, Sensitivity:92.5%, Specificity:89.0%
Ikenoyama [61]	2021	Japan	NBI+ICE+ WLE	Sensitivity:58.4%, Specificity:87.3%
Du [62]	2023	China	NF-NBI+ WLE	Accuracy:90.00%, Sensitivity:95.65%, Specificity:88.31%

IEE: image-enhanced endoscopy; ME: magnifying endoscopy; NBI: narrowband imaging; ICE: indigo carmine endoscopy; NF: near focus; WLE: white light endoscopy; AUC: area under the curve.

Tang et al. developed a deep learning-based EGC diagnostic system with an AUC of 0.888-0.951 in the validation dataset and a diagnostic rate of 100% in the video dataset. The diagnostic accuracy of the system was 93.2%, which was higher than 85.9% for senior physicians and 79.5% for junior physicians, and significantly improved physicians’ diagnostic ability [53]. Li et al. developed a CNN-based AI system to analyze gastric mucosal lesions under ME-NBI, using 386 non-cancerous and 1,702 EGC images to train the model (Inception-v3), and the results showed that the sensitivity, specificity, and accuracy of CNNs in diagnosing EGCs were 91.18%, 90.64%, and 90.91%, respectively, and there was no significant difference between CNNs and experts in the No significant difference in specificity and accuracy, but higher sensitivity. Also, the diagnostic performance of CNNs is significantly better than non-expert [54]. Horiuchi et al. validated the performance of AI in ME-NBI on 174 videos (87 cancerous and 87 non-cancerous) using 1492 cancerous and 1078 non-cancerous images for pre-training, and realized real-time diagnosis of EGC. With an AUC of 0.8684 and accuracy, sensitivity, specificity, positive predictive value, and positive predictive value of 85.1%, 87.4%, 82.8%, 83.5%, and 86.7%, respectively, the system significantly outperformed the performance of 2 experts but was slightly below the level of 1 expert, and did not show any significant difference in comparison with the remaining 8 experts [55]. A multicenter, prospective clinical trial from China confirmed the effectiveness of the AI system (ENDOANGEL-ME). With the assistance of ENDOANGEL-ME, endoscopists demonstrated higher diagnostic accuracy and sensitivity, which highlights the system’s strong capabilities in the field of EGC diagnostics [56]. The maximum magnification water immersion technique eliminates halos on endoscopic images, resulting in higher quality endoscopic images for physicians’ diagnosis. Ueyama et al. constructed a CNN computer-aided system using ME-NBI images processed by the water immersion technique at maximum magnification, which had an overall accuracy, sensitivity, specificity, positive predictive value, and negative predictive value of 98.7%, 98%, 100%, 100%, and 96.8%, respectively, with an AUC of 99% [57]. Kanesaka used 66 ME-NBI images of EGC with 60 non-cancer ME-NBI images as training data to successfully train a support vector machine (SVMLv1) model that works based on the variance vectors of the training set. Subsequently, the model was validated in a test set containing 61 ME-NBI images of EGCs with 20 non-cancer ME-NBI images, and significant results were achieved: the accuracy was up to 96.3%, the sensitivity was 96.7%, the specificity was 95% [58]. A study collected a total of 1,777 ME-NBI images of 295 patients from 3 hospitals and developed an AI system based on the VGG-19 architecture with fine-tuning. This system demonstrated an excellent performance with an AUC value of 0.808 in the internal test set and also achieved an AUC value of 0.813 in the external test set. This system is on par with senior endoscopists in terms of predictive performance and significantly outperforms junior endoscopists. With the aid of the system, the average diagnostic ability of endoscopists was significantly improved [59]. Low-grade intraepithelial neoplasia (LGIN) has a certain risk of transformation to malignancy One guideline suggests that visualized LGIN, especially lesions with clear margins, should be resected endoscopically [46]. Liu et al. retrospectively analyzed ME-NBI images of gastric tumor patients from 2 centers. Two CNN modules were developed and trained on these images, where CNN1 was trained to diagnose gastric tumors with diagnostic accuracies, sensitivity, specificity, positive predictive value, and negative predictive value of 90.8%, 92.5%, 89.0%, 89.4%, and 92.2%, respectively, and an AUC of 0.928. With the assistance of CNN1, all endoscopists had higher accuracy than the independent diagnosis [60].

Some studies have shown that indigo carmine endoscopy (ICE) improves the recognizability of depressed EGC and has a high value as an aid to diagnosis [63]. Ikenoyama et al. constructed a CNN model using 13,584 endoscopic images of 2,639 GC lesions, which included not only NBI and WLE, but also some ICEs, and the diagnostic results of the model showed sensitivity, specificity, positive predictive value, and negative predictive value of 58.4%, 87.3%, 26.0%, and 96.5%, respectively, and its The sensitivity was significantly higher than that of endoscopists, and the system analyzed the reasons for the occurrence of false-positive and false-negative diagnoses [61]. Despite the excellent ability of the ME technique to visualize diseased vessels and glandular ducts, however, in actual clinical practice, diagnosis under ME requires extensive experience and comprehensive knowledge on the part of the endoscopist, resulting in different practitioners showing significant differences in their practice and judgment. In a multicenter randomized controlled study, the accuracy of diagnosing EGCs under ME fluctuated between 40% and 85% among different endoscopists [64]. In addition the high cost and stringent technical requirements of endoscopic devices equipped with ME limit their widespread use [56]. Near focus combined narrowband imaging (NF-NBI) offers an important option for the diagnosis of lesions with simpler operation and relatively lower cost than ME-NBI [65]. In clinical practice, the guidelines strongly recommend the use of a multimodal light source in combination with a color endoscope and WLE, rather than relying on a single light source, in order to ensure accurate and efficient diagnosis and treatment [46]. Therefore Du et al. combined WLE and NF-NBI images of the same lesion into image pairs, and constructed two unimodal and three multimodal models using a total of 4201 images, 7436 image pairs and 162 videos. The results showed that the multimodal model ENDOANGEL-MM had the best diagnostic performance among the above five models, with accuracy, sensitivity, and specificity of 90.00%, 95.65%, and 88.31%, respectively, in the validated multimodal data [62].

## 3. Application of AI in determining the depth of invasion of early gastric cancer

In recent years, endoscopic submucosal dissection (ESD) has been recognized as an effective treatment for EGC [66]. Studies have shown that ESD has comparable prognostic results to surgery in the near and long term, while demonstrating its unique advantages. The technique is less invasive, has a shorter hospital stay and significantly improves the safety of the procedure. In addition, ESD has been shown to improve the quality of life of patients, providing even more significant benefits [67–70]. Indications for ESD include 1) differentiated intramucosal carcinoma without ulceration, regardless of the size of the lesion; 2) differentiated intramucosal carcinoma with ulceration, less than 3 cm in diameter; 3) undifferentiated intramucosal carcinoma without ulceration, less than 2 cm in diameter; and 4) differentiated carcinoma with submucosal infiltration of less than 500 μm, without ulceration, less than 3 cm in diameter [66]. Accurate preoperative determination of the depth of infiltration of gastric cancer plays a key role in the development of treatment strategies for gastric cancer, especially the need to determine whether the lesion has developed submucosal infiltration to determine whether the patient is able to undergo ESD. Although visualization under WLE is an effective method for determining the depth of invasion of EGCs [71], However, due to the subjective judgment of the endoscopist, it is affected by many interfering factors in the actual operation, which may lead to bias in the assessment of the depth of infiltration in some cases. Ultrasound endoscopy is also used as one of the effective examinations to determine the depth of EGC infiltration, but according to the results of the study, it does not show a more obvious advantage over conventional endoscopic images, which not only increases the length of the examination, but also imposes an additional economic burden on the patients [72,73]. Nowadays, AI has not only gained application in the detection of EGCs, but has also made some research progress in the depth of invasion of EGC (Table 3).

**Table 3.** Application of AI in determining the depth of invasion, type of differentiation, and boundary identification in early gastric cancer.

Aim: Depth of invasion				
Authors	Year	Country	Endoscopic model	Performance
Kubota [74]	2012	Japan	WLE	Mucous layer:Accuracy:68.9% Submucous layer:Accuracy:63.6 %
Bum [75]	2020	South Korea	WLE	AUC:0.887
Nagao [76]	2020	Japan	WLE+NBI+ ICE	WLE system:Accuracy:94.5% NBI system:Accuracy:94.3% Indigo AI system:Accuracy:95.5%
Zhu [77]	2018	USA	WLE	Accuracy:89.16%, Sensitivity:76.47%, Specificity:95.56%
Hong [71]	2019	South Korea	WLE	AUC:0.851
Goto [78]	2023	Japan	WLE	Accuracy:78.0%, Specificity:80.0%
Hamada [79]	2022	Japan	WLE+BLI+ ICE+LCI	Mucous layer:Accuracy:78.9% Submucous layer:Accuracy:83.8%
Kim [80]	2022	South Korea	Videos	Accuracy:83.7%, Sensitivity:82.3%, Specificity:85.8%
Wu [81]	2022	China	WLE+ME+ NBI(Videos)	Accuracy:78.57%
Chen [82]	2024	China	WLE	Accuracy:86.18%, Sensitivity:85.08%, Specificity:87.71%
**Aim: Type of differentiation**				
**Authors**	**Year**	**Country**	**Endoscopic model**	**Performance**
Wu [81]	2022	China	WLE+ME+ NBI(Videos)	Accuracy:71.43%
Ling [89]	2021	China	ME-NBI	Accuracy:83.3%
**Aim: Boundary identification**				
**Authors**	**Year**	**Country**	**Endoscopic model**	**Performance**
Ling [89]	2021	China	ME-NBI	Accuracy:82.7%-88.1%
Hu [59]	2021	China	ME-NBI	–
Kanesaka [58]	2017	Japan	ME-NBI	Accuracy:96.3%, Sensitivity:96.7%, Specificity:95%
Liu [60]	2022	China	ME-NBI	Precision:0.776, Recall:0.983, Dice:0.867
Satoko [96]	2023	Japan	WLE	Accuracy:91.7%, Sensitivity:69.9%, Specificity:94.0%
Ping [97]	2020	China	WLE+ICE	ICE system:Accuracy:85.7%, WLE system:Accuracy:88.9%

AI: artificial intelligence; WLE: white light endoscopy; NBI: narrowband imaging; ICE: indigo carmine endoscopy; LCI: linked color imaging; ME: magnifying endoscopy; AUC: area under the curve.

Kubota et al. [74] published a study of a computer-aided system for endoscopic images to assess the depth of gastric cancer infiltration. The system had an accuracy of 68.9% and a positive predictive value of 69.2% in assessing the EGC of the mucosal layer, and an accuracy of 63.6% and a positive predictive value of 68.3% in assessing the EGC of the submucosal layer. A Korean study built an AI system based on deep learning algorithms for predicting submucosal infiltration in endoscopic images of gastric tumors. They used 2,899 WLE images to fine-tune a pre-trained CNN model and validated the model with an external dataset containing 206 images. The internal test showed that the DenseNet-161 network performed well in distinguishing submucosal infiltration with an AUC of 0.887, which is consistent with the external test [75]. Sayaka et al. developed three AI systems to predict the depth of EGC invasion based on 16,557 images of 1084 patients using migration learning with ResNet50. These systems were trained with WLE, NBI, and ICE images, and the results showed that the AUC of the WLE system was 0.959, and the sensitivity, specificity, accuracy, positive and negative predictive values were 84.4%, 99.4%, 94.5%, 98.5%, and 92.9%, respectively. The three systems had similar lesion accuracies of 94.5%, 94.3%, and 95.5%, respectively, and were all effective in predicting the depth of EGC invasion [76]. Zhu et al. constructed a CNN system aimed at determining the depth of invasion of GC with high accuracy. The AUC of this system was 0.94. The sensitivity was 76.47% and the specificity was 95.56% at a threshold of 0.5. The overall accuracy was 89.16%. The positive and negative predictive values were 89.66% and 88.97%, respectively. The CNN system had significantly higher accuracy and specificity compared to endoscopists [77]. Hong Jin Yoon et al. built an optimized AI model with an AUC of 0.851 for EGC invasion depth prediction [71]. Goto et al. constructed an AI classifier for distinguishing intramucosal and submucosal GCs and designed a diagnostic method based on cooperation between AI and endoscopists. The total test images showed that the accuracy, specificity, and F1 measure of the collaborative model were 78.0%, 80.0%, and 0.776, respectively, and the accuracy using the F1 measure was higher than that using the AI or endoscopist alone [78]. The CNN model ResNet152 constructed by Hamada et al. incorporated 3508 EGC images from WLE, color imaging, BLI and ICE. Depth of invasion assessment showed sensitivity, specificity and accuracy of 84.9%, 70.7% and 78.9% for intramucosal lesions, respectively. Submucosal lesions were 85.3%, 82.4% and 83.8%, respectively [79]. Images alone may not be sufficient to fully capture the spatiotemporal dynamics during real-time endoscopy, leading to challenges in accuracy and reliability of AI trained on still images in assessing intrusion depth. In view of this, an innovative research in South Korea has realized real-time depth prediction in EGC by developing a video classifier, a model that has already performed quite impressively on still images with sensitivity, specificity, and accuracy of 82.5%, 82.9%, and 82.7%, respectively, and even more encouragingly, it works even better in video analysis, with an improved sensitivity to 82.3%, a Specificity is up to 85.8% and accuracy jumps to 83.7%.[80]. Similarly, a multicenter, prospective, real-time diagnostic study from China that included 37 EGC videos and 63 noncancerous lesions, to which 46 endoscopists from 44 hospitals in 19 provinces across China were invited, showed that the sensitivity of the system for detecting and diagnosing EGCs was 100%, significantly higher than that of the endoscopists, which was 87.13% [81].

A recent study collected a white light image dataset of 351 T1a stage and 542 T1b images to build, test, and validate the model, and the results show that the model exhibits good performance in both internal and external validation sets [82].

## 4. Application of AI in determining the differentiation type of early gastric cancer

The degree of malignancy and the incidence of lymph node metastasis were significantly higher in undifferentiated EGCs than in differentiated EGCs, which emphasizes the critical role of the type of differentiation of EGCs in determining surgical protocols and prognostic assessment [83,84]. A Meta-analysis revealed that the overall survival rates of surgical resection versus ESD resection were essentially equal in the treatment of undifferentiated EGC. However, it is noteworthy that patients resected with ESD had relatively lower rates of complete resection and showed a trend toward higher rates of recurrence [85]. As a result, treatment options for undifferentiated EGCs remain diverse. Given the guidelines [84], Accurate preoperative assessment of the degree of EGC differentiation is pivotal to the development of appropriate treatment strategies. Currently, gastroscopic pathological biopsy is the main method to determine the type of GC differentiation [86]. However, it was found that there were some differences between biopsy pathology and surgical resection pathology in determining the degree of lesion differentiation due to biopsy errors and other reasons [87,88]. Therefore, the method of determining the degree of GC differentiation preoperatively by biopsy pathology has great uncertainty. Current AI has also made some progress in determining the type of EGC differentiation (Table 3).

The AI system constructed by Wu et al. achieved an accuracy of 71.43% in predicting EGC differentiation status, a figure slightly higher than the 64.41% accuracy of endoscopists. This result fully demonstrates that this deep learning system has the potential to serve as a powerful tool for endoscopists to make EGC differentiation type judgments in clinical practice [81]. Whereas Ling et al. made significant progress in this area earlier in the year, they developed a system that was able to accurately determine the type of differentiation of a lesion with an accuracy of 83.3%, which is an excellent performance compared to experts, and successfully validated in real EGC videos [89].

## 5. Application of AI in early gastric cancer boundary recognition

In medical practice, preoperative evaluation of GC and precise definition of its boundaries are the core elements to ensure accurate judgment of GC size, effective enhancement of biopsy positivity rate, and realization of complete resection. This step is important for improving treatment outcomes and reducing the risk of recurrence [84]. There is a significant correlation between the size and magnitude of mucosal resection and potential complications such as perforation, delayed stenosis, and hemorrhage [90,91]. In addition, if the extent of the EGC is not assessed precisely enough, it may result in incomplete resection or positive margins during ESD, which in turn may lead to unnecessary additional surgeries and even increase the five-year mortality rate of patients [92,93]. Therefore, it is crucial to accurately characterize the extent of the EGC before ESD.

When ICE does not appear to be clear enough to show the margins of a lesion, the ME-NBI technique becomes an efficient and effective way to identify the entire margin of early gastric cancer [94]. It has been shown that ME-NBI does not demonstrate a significant advantage in depicting EGC margins compared to ICE, and therefore, the two methods appear to be equivalent in clinical applications [95]. This illustrates the current lack of uniformity in the precise definition of lesion boundaries, and thus, AI studies for lesion boundary determination have emerged (Table 3).

The AI system constructed by Ling et al. achieved 82.7% accuracy in differentiated EGCs and 88.1% in undifferentiated EGCs at 0.80 overlap ratio. The system was also extended with real-time delineation of EGC margins in endoscopic ME-NBI in unprocessed EGC videos [89]. Hu et al. showed that AI was not only effective in improving the diagnostic efficacy of endoscopists at different levels, but also in clarifying lesion boundaries [59]. Japanese scholars developed a computer-aided diagnostic system for endoscopists to recognize EGCs. The training set was based on the P and QGLCM feature carriers of cancerous image blocks, and the SVM was trained to delineate the cancerous blocks and compare them with the expert-delineated regions. The system had an accuracy of 96.3%, a positive predictive value of 98.3%, a sensitivity of 96.7%, a specificity of 95% [58]. Liu et al. retrospectively analyzed ME-NBI images of gastric tumor patients from two centers. Two CNN modules were developed and trained on these images, where the CNN was trained for segmentation. The average intersection between the CNN2 and the true value was 0.5837, and the precision, recall, and dice coefficient were 0.776, 0.983, and 0.867, respectively [46]. A study from Japan used 300 randomly selected endoscopic images to train the CNN and validated it with 462 cancer and 396 normal images. Thirty-eight random images were compared with images from six endoscopists, and the results showed that the system successfully detected EGCs in 387 cancer images (83.8%), with an average intersection rate of 66.5%, clearly showing the extent of the lesion, which is comparable to the expert’s results [96]. A study collected WLE and ICE images of EGC lesions to train the ENDOANGEL network and tested it on still images and ESD videos. The results showed that ENDOANGEL was 85.7% accurate on ICE images and 88.9% accurate on WLE images with an overlap ratio threshold of 0.60. In ESD videos, ENDOANGEL predicted resection margins that covered all high-grade intraepithelial neoplasia and cancer areas, which helped endoscopists to depict the extent of EGC resection [97].

## 6. Limitations and future directions

First, current studies are mainly retrospective with insufficient high-quality validation set data, which leads to the fact that the results of retrospective studies are often better than the actual clinical performance. When selecting endoscopic images for the training set, low-quality images such as bleeding, mucus, and halos are often excluded; however, these are common scenarios operated by endoscopists, and thus some of the AI model constructs are overly idealized and deviate from real clinical application scenarios. To overcome this limitation, prospective studies should be increased and more low-quality images should be included to simulate the real application environment.

Second, despite the high accuracy and specificity of many AI models, the false-positive and false-negative rates are still high. This may be due to insufficient learning of lesion and non-lesion features by the models, which needs to be improved by supplementing learning materials. Meanwhile, adding video materials and selecting clear images for each frame can greatly enrich the learning content [98].

Furthermore, most of the current multicenter studies are focused on this country, and given the differences in genes, environment, and lifestyles between Chinese and Western populations, it may be difficult to apply the geographic results broadly. Therefore, joint multinational experiments are needed to verify the broad applicability of AI.

In addition, existing studies mainly use endoscopic images from the same company, and the constructed AI model has uncertainty in recognizing images captured by different brands of endoscopes, which needs to be validated by further research. With the rapid development of endoscopic technology and the emergence of new imaging modalities, future research should focus on new high-tech endoscopic diagnosis.

Finally, most of the current studies focus on unimodal data, which may lead to the lack of completeness of AI for lesion recognition. In the future, optimal methods for multimodal data fusion should be explored to improve AI’s comprehensive recognition and learning of lesions.AI has a wide range of roles in the field of medicine, which are not limited to disease diagnosis, but also include disease prevention, personalized treatment, and disease prognosis research, and these directions will continue to be developed in depth.

## 7. Summary

The purpose of this paper is to provide a comprehensive review of research advances in artificial intelligence for endoscopic assistance in the diagnosis of EGC. It not only summarizes the detection of EGC, but also summarizes the determination of three aspects of EGC differentiation type, invasion depth, and boundary identification. Despite the wide range of aspects summarized for EGC, it was only summarized on conventional endoscopic images, for example, the study of advanced technology such as ultrasonic endoscopy for lesion diagnosis assisted by AI was not further explored in this paper, and we will focus on this aspect of inquiry in our future work.

In the field of medicine, AI has demonstrated significant advantages in several human systems, however, its application in the field of gastrointestinal tract is still in its infancy. According to current research, AI has demonstrated strong diagnostic capabilities and achieved excellent performance in human-machine comparisons. However, in the actual clinical application, continuous optimization and adjustment are still needed to achieve better auxiliary effects.

In terms of comprehensive diagnosis of EGC, AI has evolved from traditional machine learning algorithms to CNNS-based deep learning, from traditional WLE images to clearer IEE images, and from static image analysis to real-time video processing, accompanied by continuous optimization of algorithms. These advances enable AI to assist endoscopists in the multidimensional detection tasks of EGC.

Looking forward, with the inclusion of more high-quality and comprehensive research data, as well as more prospective and multicenter studies, we have reason to believe that AI will play a greater role in the diagnosis and treatment of endoscopists and bring more convenient and safer services to patients.

## Data Availability

Data sharing is not applicable to this article as no new data were created or analyzed in this study.

## Abbreviations

GC: Gastric cancer
AI: Artificial intelligence
EGC: Early gastric cancer
NBI: Narrow Band Imaging
ME: Magnifying endoscopy
BLI: Blue laser imaging
LCI: Linked color imaging
CNNs: Convolutional neural networks
WLE: White light endoscopy
DCNN: Deep convolutional neural networks
IEE: Image-enhanced endoscopy
ME: Magnifying endoscope
ME-NBI: Magnifying Endoscopy combined with Narrowband Imaging
LGIN: Low-grade intraepithelial neoplasia
ICE: Indigo carmine endoscopy
ESD: Endoscopic submucosal dissection
